# Incidence and risk factors of exercise-related knee disorders in young adult men

**DOI:** 10.1186/s12891-017-1701-3

**Published:** 2017-08-07

**Authors:** Harri K. Pihlajamäki, Mickael C. Parviainen, Hannu Kautiainen, Ilkka Kiviranta

**Affiliations:** 1 Department of Orthopaedics and Traumatology, Seinäjoki Central Hospital, Seinäjoki, Finland; 2 Faculty of Medicine and Life Sciences, University of Tampere, Tampere, Finland; 3 Mehiläinen Medical Centre, Helsinki, Finland; 4 Unit of Primary Health Care, Helsinki University Central Hospital, Helsinki, Finland; 5 Department of General Practice, University of Helsinki, Helsinki, Finland; 6 Unit of Primary Health Care, Kuopio University Hospital, Kuopio, Finland; 7 Department of Orthopaedics and Traumatology, Helsinki University Hospital and University of Helsinki, Helsinki, Finland

**Keywords:** Knee, Epidemiology, Incidence, Risk factors, Young adult, Military training, Physical activity, Disorders and injuries

## Abstract

**Background:**

Musculoskeletal disorders and injuries are common causes of morbidity and loss of active, physically demanding training days in military populations. We evaluated the incidence, diagnosis, and risk factors of knee disorders and injuries in male Finnish military conscripts.

**Methods:**

The study population comprised 5 cohorts of 1000 men performing their military service, classified according to birth year (1969, 1974, 1979, 1984, and 1989). Follow-up time for each conscript was the individual conscript’s full, completed military service period. Data for each man were collected from a standard pre-information questionnaire used by defense force healthcare officials and from all original medical reports of the garrison healthcare centers. Background variables for risk factor analysis included the conscripts’ service data, i.e., service class (A, B), length of military service, age, height, weight, body mass index (BMI), underweight, overweight, obesity, smoking habit, education, diseases, injuries, and subjective symptoms.

**Results:**

Of the 4029 conscripts, 853 visited healthcare professionals for knee symptoms during their military service, and 103 of these had suffered a knee injury. Independent risk factors for the incidence of knee symptoms were: older age; service class A; overweight (BMI 25.0–29.9 kg/m^2^); smoking habit; comprehensive school education only; and self-reported previous symptoms of the musculoskeletal, respiratory, and gastrointestinal system. The majority of visits to garrison healthcare services due to knee symptoms occurred during the first few months of military service. Knee symptoms were negatively correlated with self-reported mental and behavioral disorders.

**Conclusions:**

The present study highlights the frequency of knee disorders and injuries in young men during physically demanding military training. One-fifth of the male conscripts visited defense force healthcare professionals due to knee symptoms during their service period. Independent risk factors for the incidence of knee symptoms during military service were age at military service; military service class A; overweight; smoking habit; comprehensive school education only; and self-reported previous symptoms of the musculoskeletal system, respiratory system, or gastrointestinal system. These risk factors should be considered when planning and implementing procedures to reduce knee disorders and injuries during compulsory military service.

## Background

Knee pain and knee injuries are familiar complaints to both general practitioners and orthopedic surgeons. Knee injuries, occurring most often in young men (<30 years of age), are a major cause of pain and disability, and a widespread public health concern with regard to healthcare costs and work disability [1]. Few population-based studies of the incidence and risk factors of knee injuries in young adults are available in the literature. Physical fitness during young adulthood may affect physical fitness in older age and therefore intervention programs should target young adulthood to establish healthy habits [2].

Emergency departments in the United States treated an estimated 6.7 million knee injuries from 1999 to 2008, with an injury rate of 2.29 knee injuries per 1000 population [3]. The highest injury rate was in those 15 to 24 years of age, with males having a slightly higher injury rate than females [3]. A 2004 study conducted in a United Kingdom population reported knee pain in 15% of males 16 to 44 years of age, which resulted in disability in 16% [1]. Osteoarthritis is a primary cause of knee pain that, along with the prevalence of knee pain, increases with age in both sexes [4]. Almost 40% of all sport and leisure-time injuries are of the knee [5]. Taanila et al. [6] evaluated musculoskeletal disorders in Finnish military conscripts, and reported that injuries to the knee joint (18%) were almost as common as those to the back (20%). The vast majority of the knee injuries (91%) occurred during military exercises [6]. A large population-based study of the incidence and risk factors of knee injuries in Finnish military conscripts reported in 2013 revealed that more than 1 in 100 young adult men will be hospitalized annually due to a knee injury during their military service, and one-third of all young adult male conscripts hospitalized with knee injuries are likely to have long-term notable disability [7]. Older age and obesity are the most salient risk factors for knee injuries in military recruits [7].

Determining the risk factors for knee injuries and knee pain will facilitate the development of preventive strategies. The purpose of the present cohort study of male conscripts during their compulsory military service period was to evaluate the incidence and risk factors of knee disorders and injuries among young adult men.

## Methods

The study population comprised five cohorts of Finnish men performing their military service, classified according to birth year (1969, 1974, 1979, 1984, and 1989). We attempted to create a longitudinally representative group of young male conscripts over a time-period of 20 years by taking randomly selected cohorts of 1000 men from the Finnish population registry every fifth year starting from the year 1969. All Finnish males over 18 years of age are obligated to perform military service, and approximately 80% carry out their full service period. Chronic disease is the main reason for exemption from military service. All conscripts must pass a medical examination performed by a physician before entering the service.

A new group of conscripts enters military service twice a year, in January and in July. The mandatory military service period is a minimum of 180 days for conscripts with rank and file duties, 270 days for conscripts with special training, and a maximum of 362 days for officers and conscripts trained for particularly demanding duties. The majority of conscripts are 19 to 20 years old at the time they perform their military service. In special cases, it is possible to enter the service earlier (18 years) as a volunteer, or later, by the age of 29, when military service was formally postponed. As described in detail previously [8–10], basic training is performed by all Finnish conscripts for 8 weeks and includes various physical activities for which the intensity level is gradually increased. This 8-week basic training period comprises 135 h of physical training (17 h/week), including marching, cycling, drill training, combat training, and other training involving heavy physical loading. Combat training and marching require conscripts to carry from ~26 kg (in summer) to ~35 kg (in winter) of personal combat gear and occasionally an extra 5–20 kg of special equipment. The conscripts also perform 56 h (7 h/week) of programmed physical exercises, such as jogging, team sports, or circuit training. At the end of the 8-week training period, conscripts are expected to have attained a physical level enabling them to march or ski a distance of 15 km in one day and the same distance once again the next day carrying a full military pack weighing 25 kg and a rifle, while maintaining their fitness for battle [8–10].

After the basic training period, the amount of moderate and high-intensity programmed physical exercises is reduced to 15 h/week depending on the military branch. The intensity and volume of military training with combat gear, however, increases over the following 4 months of service (special and team training period), while the amount of moderate and high-intensity physical training is maintained at the same level in different companies. Each conscript completes approximately 450 h (19 h/week) of instructed physical training during the first 6 months of military service.

One year before entering the military service, every man or woman is evaluated and examined in a systematic health examination, usually performed by a general practitioner. Special attention is paid to self-reported symptoms and diseases detected in the health examination to assess the eligibility of each man or woman to perform military service. The service class of the conscripts, Class A (full combat or field troop training) or Class B (lightened or service training), is determined based on their fulfillment of the health requirements [9].

According to the policy of the Finnish defense forces, all conscripts are administered a standard pre-information questionnaire regarding their previous illnesses and symptoms during the first week of military service. The questionnaire charts the conscripts’ socio-economic factors, self-assessed health, and health behavior (e.g., physical activity) at baseline. The questionnaire also includes questions about school success, education level, father’s occupational group, and urbanization level of their place of residence; amount of physical exercise, prior sporting activities, membership in a sports club, and possible participation in competitive sports; significant health factors, previous sports injuries, orthopedic operations, regular medication, and any chronic disease, impairment or disability; and whether the conscript has experienced pain in seven different anatomic regions during the past month. General questions regarding health behavior include use of tobacco and alcohol, and frequency of drunkenness. Attention is paid to the individual opinion of each conscript, how he considers his health compared to age mates, and his opinion about the physical demands placed on a soldier. The questionnaire was described previously [11–14].

In addition to the health examination performed by a physician approximately 1 year before entering the military service and completing the aforementioned pre-information questionnaire, a physician re-examines the health status of every conscript during the first 2 weeks of military service to verify that all conscripts meet the health requirements for performing military service. During this visit to the garrison healthcare center, the height and weight of a conscript is measured and recorded in his file. If the medical examination reveals sequelae of knee injuries, other previous severe knee disorders, or sequelae of knee operations leading to severe disability, the conscript is discharged from military service, and thus no conscripts with this history were included in the present study. Both Class A (full combat or field troop training) and Class B (lightened or service training) conscripts [9] participated in the present study. For the present study, all original medical reports of the conscripts were scrutinized and reviewed. No data were collected from men excluded from the army before or during the service due to health reasons, and these men were not included in the study population.

Military conscripts seeking medical care must use the primary military health-care units and military hospital. Either ICD-9 or ICD-10 coding, according to the time period of the military service, was used by the military physicians to record the diagnoses. All healthcare visits of the conscripts in the present study population for any knee symptom were carefully scrutinized and reviewed to record all complaints due to disease and other disorder of the knee or knee injury.

The incidence of knee disorders and diseases or knee injuries was recorded according to the length of service and compared to possible risk factors recorded from the pre-information questionnaire. Stress fractures of the knee region were included in the knee disorders. The following information was collected from the original medical reports of each visit to the garrison healthcare center: date of the visit, symptoms, diagnosis, possible etiologic mechanism (i.e., overuse, acute trauma, other, unknown), and exemptions from duty. Body mass index (BMI) was computed as the weight in kilograms divided by the square of the height in meters (kg/m^2^). The International Classification of adult underweight, overweight, and obesity according to BMI by the World Health Organization was used to assign the conscripts to the following categories: BMI <18.50, underweight; BMI 18.50–24.99, normal; BMI 25.00–29.99, overweight; and BMI >30, obese.

The Institutional Review Board of the Finnish Defence Forces approved the study plan. The Ethics Committee of the Hospital District of Helsinki and Uusimaa approved the study (267/13/03/09).

The data are presented as means with standard deviations or as counts with percentages. The statistical significance of differences between groups was tested using a t-test or chi-square test. Time-to-event analysis was based on the product limit estimate of the cumulative incidence function. A multivariate Poisson regression model with a robust estimate of variance was used to investigate factors related to the incidence of knee symptoms. The assumption of overdispersion in the Poisson model was tested using the Lagrange multiplier test. The Stata 14.1, StataCorp LP (College Station, TX, USA) statistical package was used for the analyses.

## Results

Of the 5000 participants randomly selected for the present study using the Finnish population registry, documents concerning their military service were confirmed for 4327 men. The lack of military documents indicates that these men had not performed their military service at the time the participants were selected for the present study. Some of these men may have postponed their military service due to ongoing studies in the university or other learning institution. Others may have selected civil service instead of military service before entering the army. Some of the missing persons had been relieved of their duty to perform military service on the basis of physician examinations prior to entering the army. The aforementioned groups of men lack military service documents because they had not entered the military service at the time we selected the cohorts. Of the 4327 men with documentation, 298 were excluded because they selected civil service instead of the army after entering military service or they were discharged for health reasons. Altogether, data were collected for analysis from 4029 conscripts. Of these 4029 conscripts, 853 (21.2%) visited healthcare professionals (physicians or nurses) for knee symptoms at least once during their military service period. These 853 conscripts visited the military medical service facilities for knee symptoms a total of 2118 times during their service period. Of these 2118 visits to seek medical advice, 232 (11%) were for a recent knee injury (95% CI: 9.6–12.3). For the whole study population of 4029 men, the total number of visits to the garrison healthcare center was 32,510.

The demographic and clinical characteristics of conscripts before entry into the military service are shown in Table 1. Finnish conscripts assigned to Class A military service were at greater risk for knee disorders and injuries during their compulsory military service period. Conscripts with a history of mental and behavioral disorders before entering the military service had a lower risk for knee disorders and injuries during their military service (Table 1). Of conscripts with mental symptoms, 188 (5.0%) served as Class A soldiers and 90 (29.7%) as Class B soldiers (*p* < 0.001).

**Table 1:** Demographic and clinical characteristics of conscripts before entry into the military service stratified by knee symptoms during military service

Characteristic	Knee symptoms during military service		
	Yes *N* = 853	No *N* = 3176	*P*-value
Age at military service, year, mean (SD)	19.2 (1.0)	19.3 (1.2)	0.094
Military Service Class A, n (%)	811(95.1)	2915 (91.8)	<0.001
BMI, mean (SD)	23.1 (3.7)	23.3 (3.8)	0.26
Smoking (cigarettes/day), n (%)			0.004
0	458 (62.6)	1834 (37.4)	
1–9	62 (8.5)	263 (9.7)	
10–19	150 (20.5)	474 (17.4)	
≥ 20	61 (8.3)	151 (5.5)	
Comprehensive school only, n (%)	499 (58.6)	1846 (58.5)	0.96
High physical activity, n (%)	283 (38.8)	1025 (37.6)	0.56
Diseases and injuries detected by military physicians, n (%)			
Mental or behavioral disorders	9 (1.1)	169 (5.3)	<0.001
Diseases of the eye and adnexa	219 (25.7)	883 (27.8)	0.22
Diseases of the respiratory system	142 (16.6)	450 (14.2)	0.069
Diseases of the skin and subcutaneous tissue	46 (5.4)	184 (5.8)	0.65
Diseases of the musculoskeletal system	84 (9.8)	264 (8.3)	0.16
Injuries	103 (12.1)	315 (9.9)	0.067
Flatfoot deformity, n (%)	14 (1.6)	78 (2.5)	0.16
Subjective symptoms reported in official questionnaire before entering military service, n (%)			
Symptoms of the musculoskeletal system	93 (10.9)	261 (8.2)	0.014
Symptoms of the respiratory system	195 (22.9)	646 (20.3)	0.11
Symptoms of the gastrointestinal system	25 (2.9)	63 (2.0)	0.093
Mental symptoms	24 (2.8)	117 (3.7)	0.22

**Fig. 1:**
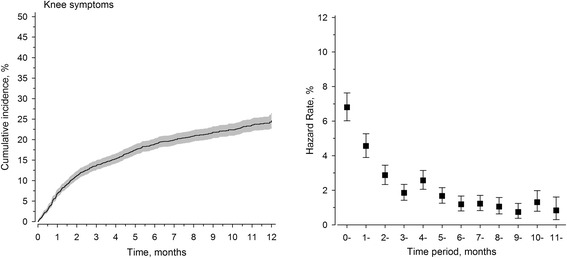
Cumulative incidence and hazard rate of knee symptoms during military service

The cumulative incidence [24.6% (95% CI: 22.9 to 26.4)] of knee symptoms is shown in Fig. 1. Peak incidence occurred during the first few months of military service, and the number of visits decreased over time in the service. In the univariate analysis, the groups with or without knee symptoms differed in age, military service class, smoking habit, mental disorders, and previous injuries, as well as asthma condition and other respiratory and mental symptoms (Table 1). In the multivariate analysis, age at military service, military service class A, overweight (BMI 25.0–29.9 kg/m^2^), smoking habit, comprehensive school only, self-reported previous symptoms of the musculoskeletal system, self-reported previous symptoms of the respiratory system, and self-reported previous symptoms of the gastrointestinal system were independent risk factors for the incidence of knee symptoms during military service (Table 2).

**Table 2:** Poisson regression model for number of knee disorders or injuries

	IRR (95% CI)	*P*-value
Age	0.92 (0.87–0.96)	<0.001
Military Service Class A	1.29 (1.05–1.57)	0.014
BMI (Body Mass Index)		
< 25.0 kg/m^2^	1.00 (Reference)	
25.0–29.9 kg/m^2^	1.15 (1.03–1.30)	0.016
≥ 30 kg/m^2^	0.98 (0.80–1.20)	0.87
High physical activity	0.99 (0.90–1.10)	0.91
Smoking (cigarettes/day)		
0	1.00 (Reference)	
1–9	1.24 (1.06 to 1.45)	0.009
10–19	1.37 (1.21 to 1.55)	<0.001
≥ 20	1.35 (1.11 to 1.64)	<0.001
Comprehensive school only	1.10 (1.00–1.22)	0.046
Injuries	1.07 (0.93–1.23)	0.34
Symptoms of the musculoskeletal system	1.59 (1.39–1.82)	<0.001
Symptoms of the respiratory system	1.17 (1.05–1.30)	0.005
Symptoms of the gastrointestinal system	1.67 (1.31–2.12)	<0.001
Mental symptoms	0.82 (0.64–1.06)	0.13

## Discussion

The main finding of the present study was that 20% of the male conscripts visited defense force healthcare professionals at least once during their compulsory military service period for symptoms related to a knee disorder or injury. Thus, a total of 853 of 4029 young adult males sought medical advice regarding knee symptoms at least once during their military service. In a previous study of knee injuries requiring hospitalization of conscripts, the incidence rate was 11 cases per 1000 person-years, indicating that at least 1 of 100 young adult males is hospitalized each year due to knee injury during their conscription [7]. In the present investigation, we also calculated all conscript visits to healthcare professionals due to any knee symptoms, not only knee injuries leading to hospitalization of the patient. The results of the present study highlight the frequency of knee disorders and injuries in young male adults during physically demanding military training, and indicate the need for preventive measures to diminish knee disorders and injuries during military service.

The peak incidence of conscript visits to seek medical advice due to knee symptoms in military healthcare facilities occurred during the first few months of military service, which might be due to the fact that all Finnish conscripts start their basic training with 8 weeks of physical activity that gradually increases in intensity over time.

According to the findings of the present study, overweight (BMI 25.0–29.9 kg/m^2^) was a risk factor for the incidence of knee symptoms during military service. Previous studies of military populations reported that overweight is a significant risk factor for musculoskeletal injuries in general [1, 9, 15]. Moreover, a study of Israeli conscripts reported an association between overweight and lower extremity joint conditions [16]. In another study of 17 year-old adolescents, obesity was associated with knee pain [17]. Weight gain is reportedly associated with more severe knee pain, and weight loss is reported to relieve knee symptoms [18]. Frilander et al. [19] reported in a study of Finnish conscripts that becoming severely overweight during the follow-up after military service is associated with knee pain, and becoming severely obese predicts the development of functional limitations. According to that study, traumatic knee problems during military service partly mediate the effect of obesity on functional limitations [19].

The present study also revealed cigarette smoking to be an independent risk factor for the incidence of knee symptoms during military service. A previous study reported the prevalence of cigarette smoking among Finnish conscripts and demonstrated that the prevalence of a smoking habit decreased from 42% to 34% during the period from 1999 to 2010 [20]. According to a population-based study on 58-year-old persons, however, smoking is not associated with knee pain [21]. In addition, a recent meta-analysis of risk factors for knee osteoarthritis in older adults revealed no association between smoking and the onset of knee osteoarthritis [22]. No population-based studies have examined the association of smoking and knee pain among younger adults without knee osteoarthritis.

Age at military service and previous education comprising only comprehensive school were also independent risk factors for the incidence of knee symptoms during military service. Moreover, conscripts assigned to Class A military service had a statistically higher risk of knee symptoms. One potential explanation for this is that the military training for Class A conscripts is physically more demanding than that for conscripts serving as Class B conscripts. Mental symptoms and psychiatric diagnoses in either or both of the pre-information questionnaires and during military service correlated negatively with knee symptoms. Conscripts with mental or behavioral disorders are often entered into Class B service and thus have lighter physical service and training than Class A conscripts. According to the findings of the present study, only 5.0% of conscripts with mental symptoms served as Class A conscripts and 30% as Class B conscripts. Self-reported previous symptoms of the musculoskeletal system, self-reported previous symptoms of the respiratory system, and self-reported previous symptoms of the gastrointestinal system were also independent risk factors for the incidence of knee symptoms during military service.

This study has several strengths. First, this study includes a large study population of 4029 male conscripts from five different age cohorts in a nation where military service is compulsory for male citizens. Second, because military service is compulsory in Finland, the male conscripts represent young Finnish adults that were considered healthy enough to enter military service based on a complete examination performed by a physician. The findings of the present study regarding knee injuries in conscripts are thus generalizable to the young adult male population. Compulsory military service is a useful indicator of the response to a progressive physical exercise regimen by young adults in the general population. Third, importantly, conscripts are required to use the primary military health-care units and Central Military Hospital for all medical treatment. Fourth, the data analyzed in the present study were collected from original pre-military service medical records as well as the military service medical records and an official standard pre-information questionnaire regarding previous illnesses and symptoms completed by every conscript.

Only males were included in this study, which may limit interpretation of the findings. Further, in the older age cohorts, some medical reports were manually documented, and the notes were not standardized. From the original study population of 5000 subjects, medical reports were obtained for 4327. Most of the missing reports were for those born in 1989, because some of these men were currently serving or were about to serve in the near future, and thus service data were not yet available for inclusion in the present study. Due to the rapid recovery and mild nature of the majority the knee symptoms of the conscripts, the exact diagnosis of the knee disorder was seldom available in the medical records. Thus, classification of the patients based on the exact diagnosis was not possible; instead, we classified the knee disorders as traumatic or non-traumatic.

## Conclusions

The results of the present study highlight the frequency of knee disorders and injuries in young male adults during physically demanding military training. One-fifth of the male conscripts visited defense force healthcare professionals due to knee symptoms during their service period. Based on the findings of the present study, age at military service, military service class A, overweight (BMI 25.0–29.9 kg/m^2^), smoking habit, comprehensive school only, self-reported previous symptoms of the musculoskeletal system, self-reported previous symptoms of the respiratory system, and self-reported previous symptoms of the gastrointestinal system are independent risk factors for the incidence of knee symptoms during military service. These risk factors should be taken into account when planning and implementing procedures to reduce knee disorders and injuries during compulsory military service.

## Abbreviations

BMI: Body Mass Index
